# Copy number variants prioritization after array-CGH analysis – a cohort of 1000 patients 

**DOI:** 10.1186/s13039-015-0202-z

**Published:** 2015-12-30

**Authors:** Isabel Marques Carreira, Susana Isabel Ferreira, Eunice Matoso, Luís Miguel Pires, José Ferrão, Ana Jardim, Alexandra Mascarenhas, Marta Pinto, Nuno Lavoura, Cláudia Pais, Patrícia Paiva, Lúcia Simões, Francisco Caramelo, Lina Ramos, Margarida Venâncio, Fabiana Ramos, Ana Beleza, Joaquim Sá, Jorge Saraiva, Joana Barbosa de Melo

**Affiliations:** Laboratório de Citogenética e Genómica – Faculdade de Medicina, Universidade de Coimbra, Pólo Ciências da Saúde, Sub-Unidade 1 - Piso 2, Azinhaga de Santa Comba, 3000-354 Coimbra, Portugal; CIMAGO – Centro de Investigação em Meio Ambiente, Genética e Oncobiologia, Faculdade de Medicina, Universidade de Coimbra, Coimbra, Portugal; Faculdade de Medicina, Universidade de Coimbra, Coimbra, Portugal; CNC, IBILI – Faculdade de Medicina, Universidade de Coimbra, Coimbra, Portugal; Laboratório de Citogenética, Hospital Pediátrico de Coimbra, Coimbra, Portugal; Laboratório de Bioestatística e Informática Médica, IBILI – Faculdade de Medicina, Universidade de Coimbra, Coimbra, Portugal; Serviço de Genética Médica, Hospital Pediátrico – Centro Hospitalar e Universitário de Coimbra, Coimbra, Portugal

**Keywords:** Array comparative genomic hybridization (array-CGH), Copy number variation (CNV) classification, Intellectual disability, Multiple congenital anomalies, Learning difficulties, Autism spectrum disorders

## Abstract

**Background:**

Array-based comparative genomic hybridization has been assumed to be the first genetic test offered to detect genomic imbalances in patients with unexplained intellectual disability with or without dysmorphisms, multiple congenital anomalies, learning difficulties and autism spectrum disorders.

Our study contributes to the genotype/phenotype correlation with the delineation of laboratory criteria which help to classify the different copy number variants (CNVs) detected. We clustered our findings into five classes ranging from an imbalance detected in a microdeletion/duplication syndrome region (class I) to imbalances that had previously been reported in normal subjects in the Database of Genomic Variants (DGV) and thus considered common variants (class IV).

**Results:**

All the analyzed 1000 patients had at least one CNV independently of its clinical significance. Most of them, as expected, were alterations already reported in the DGV for normal individuals (class IV) or without known coding genes (class III-B). In approximately 14 % of the patients an imbalance involving known coding genes, but with partially overlapping or low frequency of CNVs described in the DGV was identified (class IIIA). In 10.4 % of the patients a pathogenic CNV that explained the phenotype was identified consisting of: 40 class I imbalances, 44 class II *de novo* imbalances and 21 class II X-chromosome imbalances in male patients. In 20 % of the patients a familial pathogenic or potentially pathogenic CNV, consisting of inherited class II imbalances, was identified that implied a family evaluation by the clinical geneticists.

**Conclusions:**

As this interpretation can be sometimes difficult, particularly if it is not possible to study the parents, using the proposed classification we were able to prioritize the multiple imbalances that are identified in each patient without immediately having to classify them as pathogenic or benign.

## Background

Microarray-based comparative genomic hybridization (array-CGH), also called chromosomal microarray or molecular karyotyping, allows the possibility to screen the whole genome at once and with high resolution. It is currently assumed that array-CGH should be the first genetic test offered to detect genomic imbalances in patients with intellectual disability (ID) with or without dysmorphisms, multiple congenital anomalies (MCA), learning difficulties and autism spectrum disorders (ASD) [1, 2]. As array-CGH allows the detection of imbalances below the 5–10 Mb resolution level of conventional cytogenetics, the average diagnostic yield can be up to 10 % higher [3–7]. It also allows the detection of a large number of Copy Number Variants (CNVs) in these patients as well as in healthy individuals, which poses a great challenge in the interpretation of the results [8]. In addition to this increase in the detection of CNVs, the use of array-CGH in large cohorts of patients with ID, ASD and MCA has led to the identification of novel microdeletion and microduplication syndromes [9, 10].

In this study we report the frequency of imbalances, their inheritance and conclusions of a cohort of 1000 patients with ID, ASD, MCA and learning disabilities studied by array-CGH. We propose a classification of CNVs into five different classes as a way to prioritize the multiple imbalances detected in each patient, helping not only the organization and interpretation of the results, but also in those cases where parents were unwillingly to be tested or were unavailable, the elaboration of a more comprehensive report.

## Results

A cohort of a 1000 patients with ID, MCA and/or ASD was studied by array CGH using an oligonucleotide platform. After array analysis, detected CNVs were classified into 5 different classes (Table 1): class I - deletions or duplications in a region associated with a microdeletion or a microduplication syndrome; class II - deletions or duplications in a region not reported in the Database of Genomic Variants (DGV) and involving known coding genes; class IIIA- deletions or duplications reported in low frequency in DGV or that do not totally overlapped with the ones reported, involving known coding regions; class IIIB - deletions or duplications reported in low frequency in DGV or that do not totally overlapped with the ones reported, without involving known coding genes; class IV - deletions or duplications reported in normal subjects in DGV or that are considered common variants.

**Table 1:**
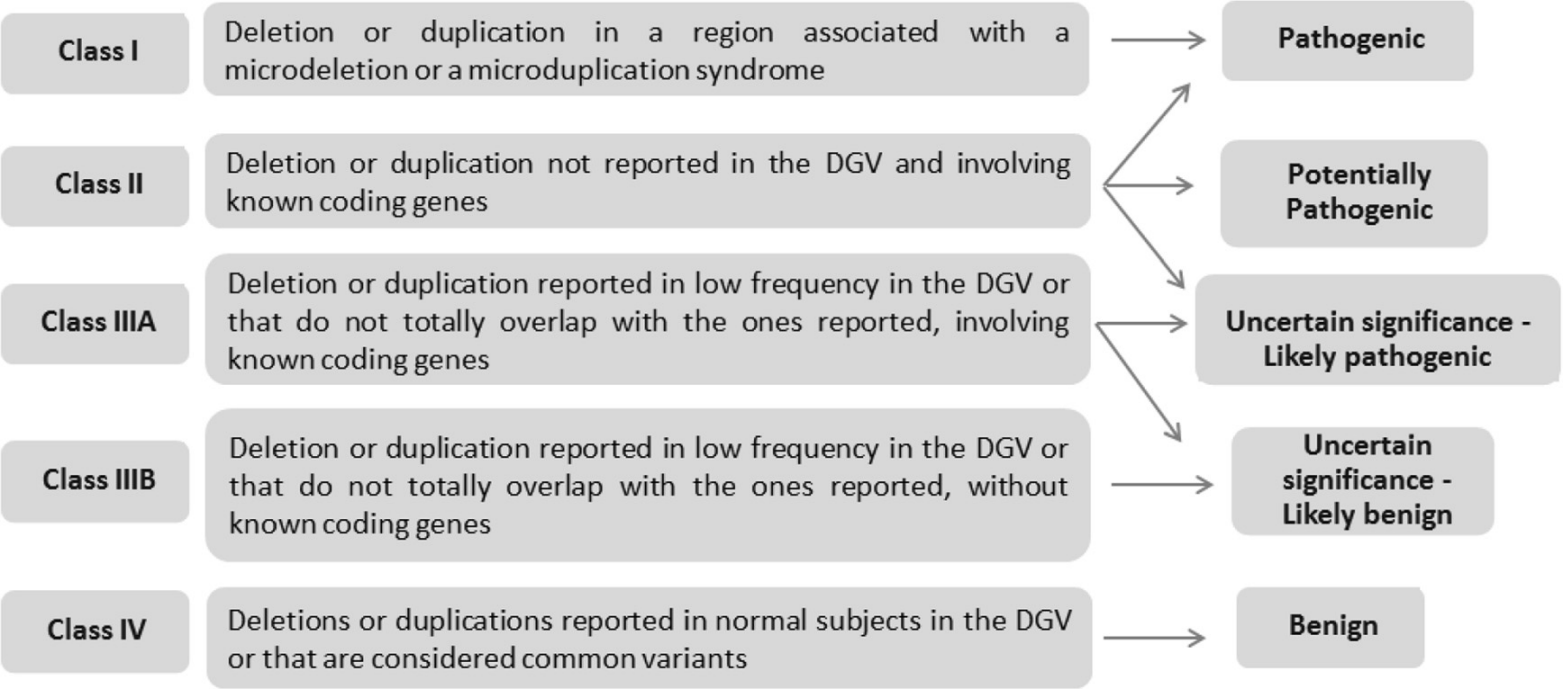
Class definition of CNVs depending on its clinical significance

When a class I or class II CNV was found, the study of the parents was requested in order to ascertain its origin and delineate the strategy for family management and future prenatal diagnosis. For cases where a IIIA CNV was identified, decision whether or not to pursue with parental evaluation was left to the clinicians. For class IIIB and class IV CNVs no study of parents was needed, and a normal array-CGH result was issued. Since class IIIB CNVs at present do not have genes reported, no causality was attributed, but the information was included in a summary table in the report, to allow a future revision, if genes or regulatory regions are later described.

There were cases where the origin of the imbalances was not possible to determine, either because of unwillingness of both parents to be evaluated, in situations of adoption or in monoparental cases in which the available progenitor was not a carrier of the proband’s CNVs.

**Fig. 1:**
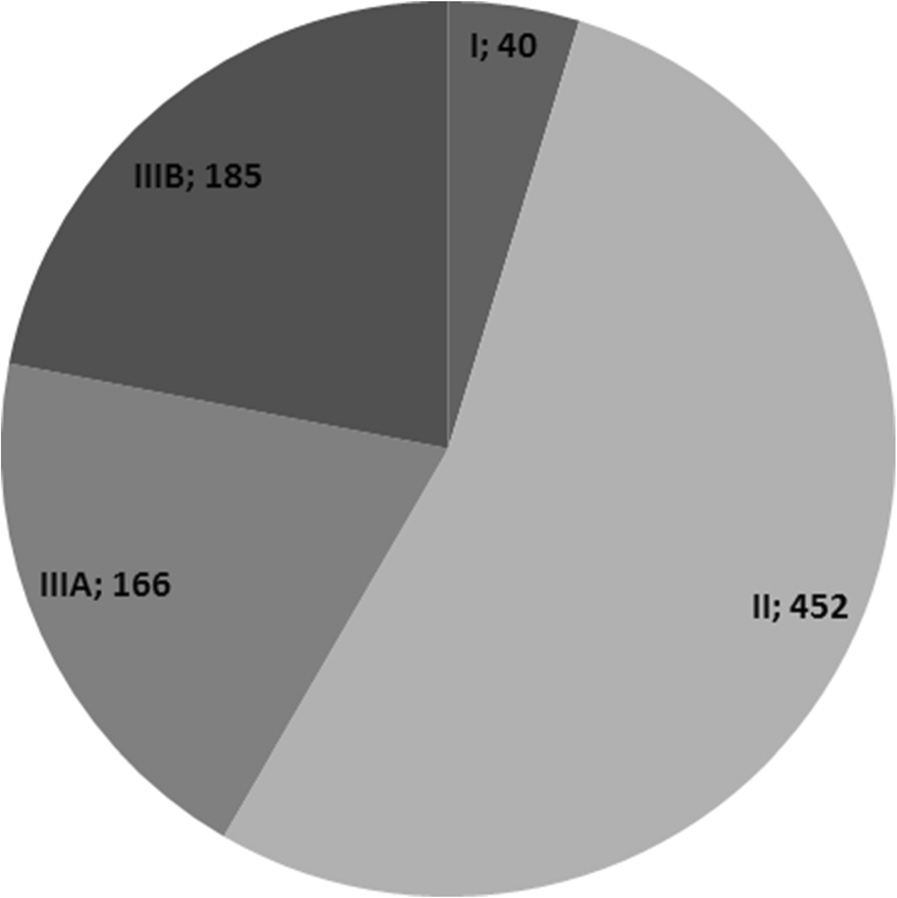
Distribution of the 843 CNVs identified in 611 of the 1000 cases by classes I to IIIA: 40 imbalances belong to class I (4.8 %), 452 to class II (53.6 %), 166 to class IIIA (19.7 %) and 185 to class IIIB (21.9 %)

In our cohort we identified at least one class IV CNV in each patient. As they are considered benign copy number variations present in normal individuals, like the results by Choucair and collaborators [11], class IV imbalances were disregarded for the frequency evaluation. In this study group, 489 patients only had class IV imbalances. Excluding class IV CNVs, we identified a total of 843 CNVs in the remaining 611 cases: 40 as class I (4.8 %), 452 as class II (53.6 %), 166 as class IIIA (19.7 %) and 185 as class IIIB (21.9 %) (Fig. 1).

**Fig. 2:**
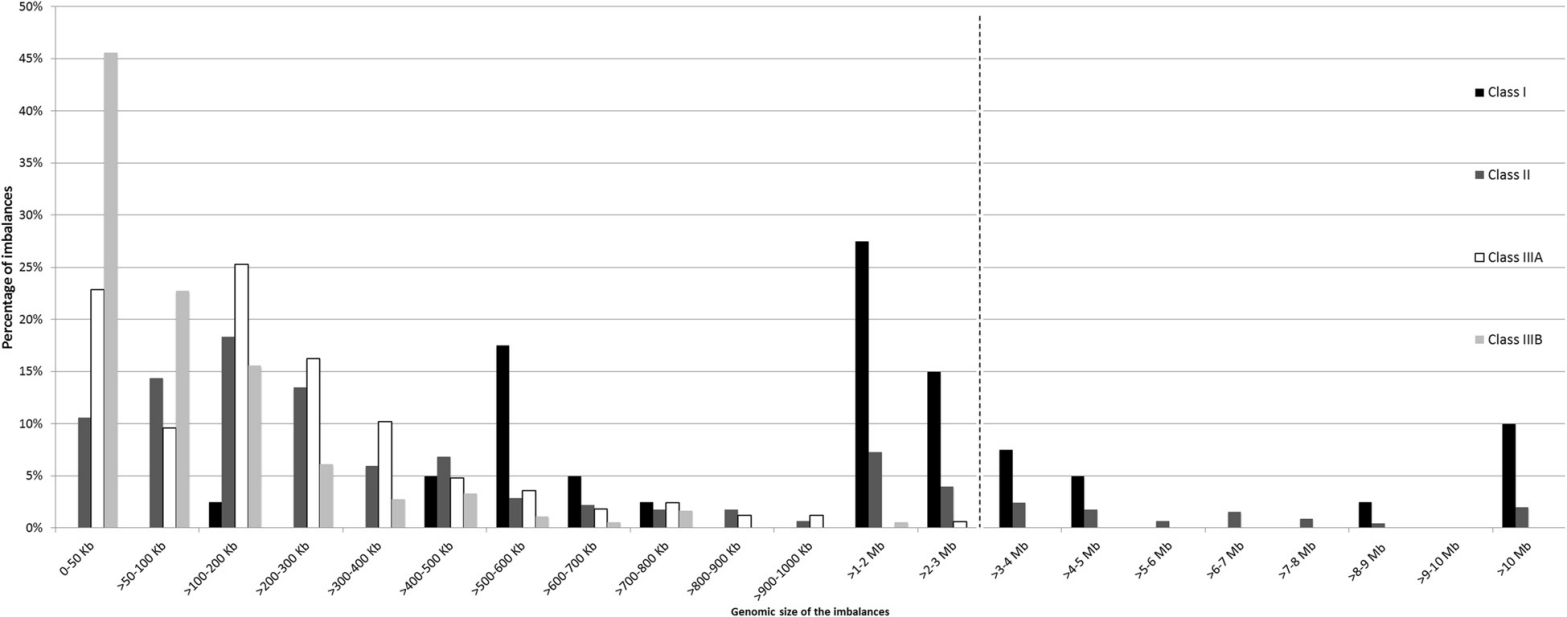
Distribution of all the imbalances according to the genomic size. As the genomic size of the imbalance increases, the number of imbalances from the four classes reduces. Above 3 Mb in size only imbalances belonging to classes I and II are observed. Class IV imbalances are not represented as they are considered benign copy number variations. There is an association with statistical significance (*χ* ^2^(1) = 40.591; p < 0.001) between classes I and II and sizes above 3 Mb and between classes IIIA and IIIB and sizes below 3 Mb – dashed line

The distribution of all the imbalances from the different classes according to genomic size was evaluated (Fig. 2). From the figure analysis we can observe that as the genomic size of the imbalance increases, the probability to find imbalances from the four classes reduces. Above 3 Mb in size we only found imbalances belonging to class I and II. There is an association with statistical significance (*χ*^2^(1) = 40.591; p < 0.001) between class I and II and sizes above 3 Mb, and between class IIIA and IIIB and sizes below 3 Mb.

Class I imbalances (Table 2) were present in 40 patients: twenty-two deletions and sixteen duplications, a triple X and a Klinefelter syndrome which were confirmed without other clinical significant imbalances present. Fifteen imbalances were *de novo*, two maternal, four paternal and in nineteen cases the inheritance was not possible to determine. Table 3 contains the list of all the syndromes identified and the number of cases within each one. Of the 40 imbalances, 42.5 % had between 1–3 Mb in size, eleven cases (27.5 %) had a genomic size between 1-2 Mb and six (15 %) an imbalance with 2-3 Mb, with the remaining distributed by different genomic ranges (Fig. 2). The smallest class I imbalance detected corresponds to a 184 kb deletion in 8q22.2 involving Cohen syndrome gene, *COH1*, and the largest, disregarding the triple X and Klinefelter cases, corresponds to a 30 Mb deletion at the short arm of chromosome 5, involving the Cri du Chat region. In 38 % of the cases the imbalances were *de novo* and in 48 % it was not possible to ascertain the family inheritance. From the available data, it was shown that chromosome 3q29 deletion and the 17q12 duplication were similarly observed in the mothers, while the chromosomes 3q29 duplication syndrome, 16p11.2 deletion syndrome and 22q11.2 duplication and deletion syndromes were observed similarly in the male progenitors.

**Table 2:** Class I imbalances distributed according to the type of imbalance and inheritance

Imbalance	Deletion	Duplication	Klinefelter	Triple X	
Inheritance					
*De novo*	8	5	1	1	15
Maternal	1	1	0	0	2
Paternal	2	2	0	0	4
Unknown	11	8	0	0	19
	22	16	1	1	40

**Table 3:** List of all the syndromes identified (class I imbalances) and the number of cases

Syndrome	Chromosome	OMIM ID	Number of cases
Chromosome 1q21.1 deletion syndrome	1q21.1	612474	3 na
Chromosome 1q21.1 duplication syndrome	1q21.1	612475	1 na
Chromosome 3q29 deletion syndrome	3q29	609425	1 mat
Chromosome 3q29 duplication syndrome	3q29	611936	2 (1pat, 1na)
Wolf-Hirschhorn syndrome	4p16.3	194190	1 dn
Cri du Chat syndrome	5p	123450	1 na
Williams-Beuren region duplication syndrome	7q11.23	609757	2 (1dn, 1na)
Cohen syndrome	8q22.2	216550	1 na
Chromosome 9p deletion syndrome	9p	158170	1 dn
Kleefstra syndrome	9q34.3	610253	1 na
Chromosome15q11q13 duplication syndrome	15q11q13	608636	1 na
Chromosome 15q13.3 deletion syndrome	15q13.3	612001	1 na
Chromosome 16p11.2 deletion syndrome	16p11.2	611913	5 (1pat, 2dn, 2na)
Chromosome 16p11.2 duplication syndrome	16p11.2	614671	3 (1dn, 2na)
Smith –Magenis syndrome	17p11.2	182290	1 na
Potocki-Lupski syndrome	17p11.2	610883	2 dn
Chromosome 17q12 duplication syndrome	17q11.2	614526	2 (1mat,1na)
Chromosome 19q13.11 deletion syndrome	19q13.11	613026	1 dn
Chromosome 22q11.2 deletion syndrome, distal	22q11.2	611867	5 (1pat, 3dn, 1na)
Chromosome 22q11.2 duplication syndrome	22q11.2	608363	3 (1pat, 1dn, 1na)
Triple X	X		1dn
Klinefelter	X		1dn

*mat* maternal origin, *pat* paternal origin, *dn de novo*, *na* inheritance not available

We detected 452 class II imbalances present in 340 cases, since some patients had multiple imbalances classified in this class. Of the 452 alterations, 191 were deletions (42.25 %), 255 duplications (56.42 %), two were X-chromosome triplications (0.44 %) and the remaining four were tetrasomies (0.89 %) (Table 4). Considering the totality of class II imbalances, 70 % had a genomic size between 1.9 kb, that corresponds to the size of the smallest imbalance detected (a deletion), and 500 kb. 15 % of the cases had an imbalance between 1–5 Mb, with the remaining distributed by different genomic ranges, as observed in Fig. 2.

**Table 4:** Class II imbalances distributed according to the type of imbalance and inheritance

Imbalance	Deletion	Duplication	Triplication	Tetrasomy	
Inheritance					
*De novo*	30	13	0	1	44
Maternal	38	74	0	2	114
Paternal	38	48	0	0	86
Maternal and paternal	1	0	0	1	2
Unknown	84	120	2	0	206
	191	255	2	4	452

**Fig. 3:**
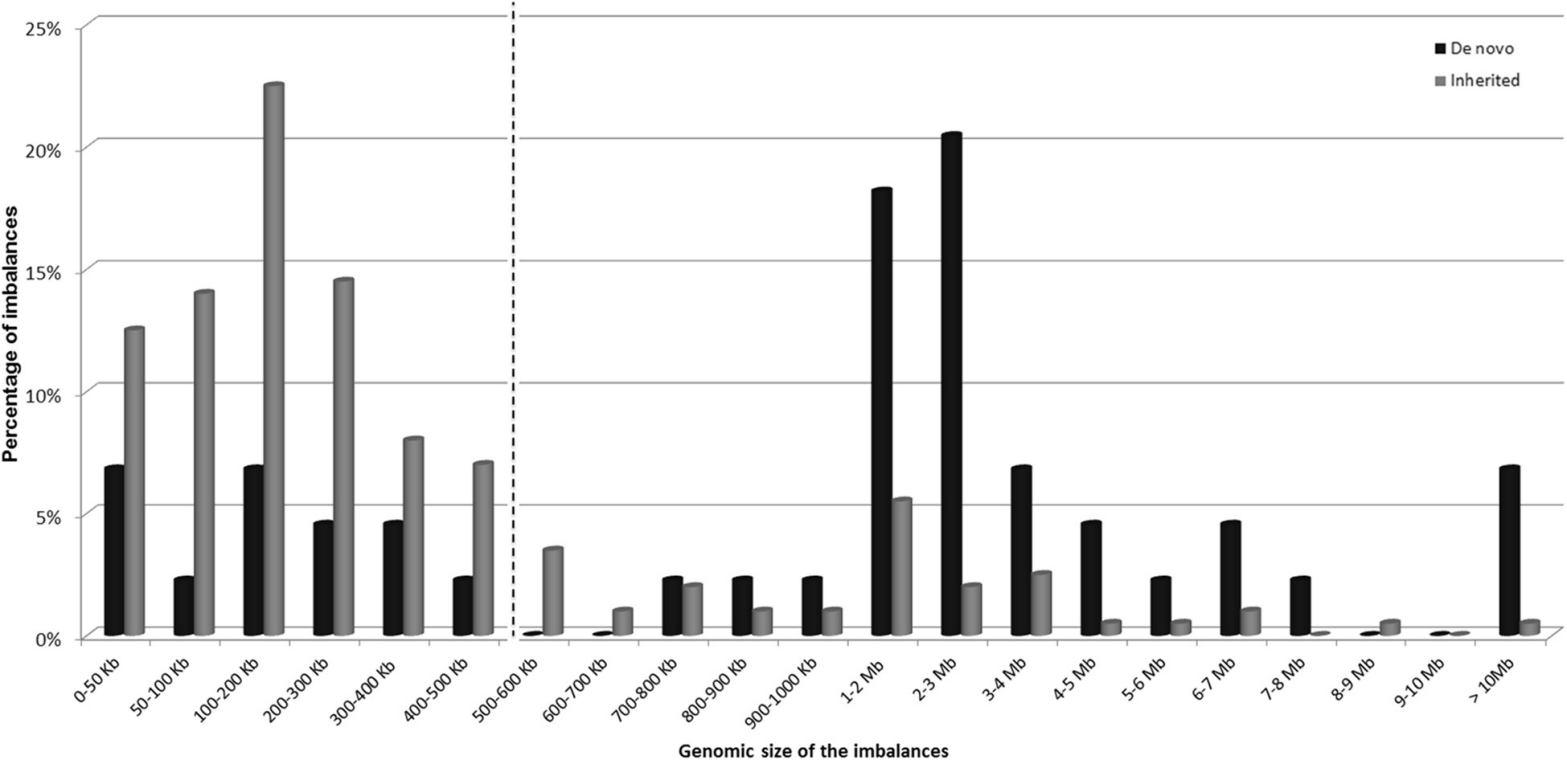
Class II *de novo* and inherited imbalances. In 82 % of the inherited CNVs had a genomic size ranging from 1.985 kb to 600 kb, against 28 % of the de novo, while 51 % of the de novo imbalances had a genomic size ranging from 1 to 5 Mb. The higher the genomic size of the imbalance, the lower the probability of being inherited. Our data show a significant association (*χ* ^2^(1) = 44.456; p < 0.001) between *de novo* imbalances and sizes above 500 kb and between inherited imbalances and sizes below 500 kb – dashed line

Analyzing class II imbalances in respect to inheritance pattern, there was a clear difference considering the distribution of inherited vs *de novo* imbalances: 82 % of the inherited CNVs had a genomic size ranging from 1.985 kb to 600 kb, against 28 % of the *de novo*, while 51 % of the *de novo* imbalances had a genomic size ranging from 1 to 5 Mb. The higher the genomic size of the imbalance, the lower the probability of being inherited (Fig. 3). Our data show a significant association (*χ*^2^(1) = 44.456; p < 0.001) between *de novo* imbalances and sizes above 500 kb and between inherited imbalances and sizes below 500 kb.

One hundred and sixty-six class IIIA imbalances were identified in 137 different patients: sixty deletions (36.15 %), 103 duplications (62.05 %) and three amplifications (1.8 %). To date, only one patient whose parents were studied revealed to have a *de novo* class IIIA imbalance. In the others there was a similar proportion of maternal and paternal inheritance (Table 5). The maximum genomic size of imbalances in this class was 2.5 Mb, with 84 % of the imbalances ranging from 1.9 kb and 400 kb (Fig. 2).

**Table 5:** Class IIIA imbalances distributed according to the type of imbalance and inheritance

Imbalance	Deletion	Duplication	Amplification	
Inheritance				
*De novo*	0	1	0	1
Maternal	10	9	0	19
Paternal	10	6	0	16
Unknown	83	44	3	130
	103	60	3	166

We identified 185 class IIIB imbalances, fifty-six deletions (30.3 %), thirty duplications (16.2 %) and ninety-nine variations (53.5 %), ranging from 284 bp to 1.4 Mb, with 84 % of them having between 284 bp-200 kb.

**Fig. 4:**
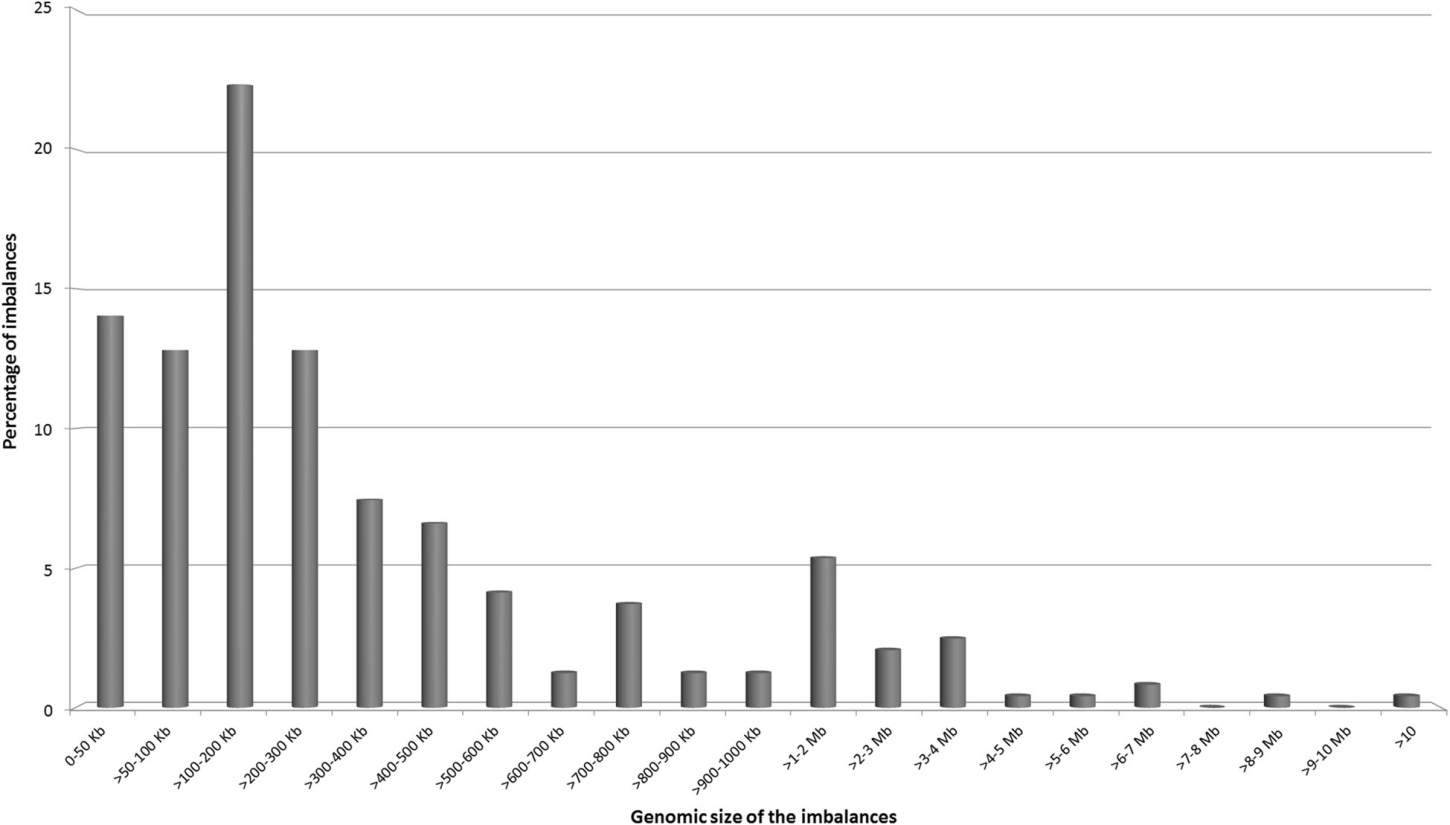
Inherited CNVs distribution according to genomic size. 76 % of all the inherited CNVs identified were smaller than 500 kb

In the studied cohort of patients, we concluded that the great majority, 76 % of all the inherited CNVs identified were smaller than 500 kb, as Fig. 4 illustrates.

## Discussion

Array-CGH has proven to be an important tool not only in the detection of submicroscopic chromosomal imbalances and delineation of structural imbalances but also in the establishment of genotype/phenotype correlation. All of the 1000 cases studied in this cohort presented ID with or without dysmorphisms, learning difficulties, MCA or ASD. All of them had imbalances detected by array-CGH. Overall, there were 843 imbalances (class I-III) in 611 of the 1000 cases (Fig. 1). Imbalances classified as class IV are not discussed as they correspond to benign CNVs. In order to define the relevance of the identified imbalances and their correlation with the identified phenotypes we classified them into five classes:

### Class I - Deletion or duplication in a region associated with a microdeletion or a microduplication syndrome

Of the 1000 patients, forty (4 %) fitted into this category with more deletions compared to duplications. In a study by Shoukier and collaborators, in 342 patients, 42 (12 %) had an imbalance that corresponded to a known microdeletion or microduplication syndrome, with a higher proportion of deletions to duplications (33 vs 9) [12]. The most common findings in our cohort were imbalances involving 16p11.2 and 22q11.2 microdeletion/duplication syndromes region, interestingly the same result as recently reported by Ahn et al, 2013 [13]. In the cases where pattern of inheritance was studied, as expected, most of them were *de novo*. One of these cases had a *de novo* 19q13.11q13.12 deletion that provides evidence for the involvement of *UBA2* gene haploinsuffiency in cutis aplasia [14].

Of the six inherited imbalances, three were deletions in chromosomes 3q29, 16p11.2 and 22q11.2, and three were duplications, in 3q29, 17q12 and 22q11.2. The 3q29 deletion and the 17q12 duplication were maternally inherited, while the 3q29 and 22q11.2 duplications and the 16p11.2 and 22q11.2 deletions were paternal in origin. In the results by Shoukier and collaborators, half of the 42 imbalances were *de novo*, 9 were maternally inherited, with 4 imbalances on the X-chromosome, and in 12 inheritance was unknown [12].

Concerning inherited CNVs, the difficulties in interpretation are due to: 1) incomplete penetrance; 2) phenotypic variability; and 3) lack of clinical evaluation of the carrier progenitor. The chromosome 16p11.2 deletion was inherited from a healthy father, which is commonly reported in the literature and in agreement with the variable clinical outcome associated with this imbalance that can include a normal phenotype [12, 15]. Concerning chromosome 22q11.2 imbalances, the father with the 22q11.2 duplication is healthy, having however only completed the basic scholarship but is currently not able to read or write and his ID is similar to his daughter. The chromosome 22q11.2 deletion is inherited from the paternal grandmother, and the proband is the only one with phenotypic features. These cases clearly show the inter and intra-familial phenotypic variability associated with this imbalance [16]. The 3q29 deletion was inherited from the mother without ID but that had learning difficulties, and actually presents speech difficulties and psychiatric disease. The 3q29 duplication and the 17q12 duplication were inherited from healthy progenitors that have only completed basic scholarship.

The inherited cases posed a delicate problem, in terms of future pregnancies, in healthy parents that had an apparently normal phenotype, even though many of them had only completed basic schooling.

Class I imbalances are considered pathogenic and molecular/conventional cytogenetics-FISH study of the progenitors is recommended, namely to exclude a balanced rearrangement (Table 1).

### Class II - Deletion or duplication not reported in the DGV and involving known coding genes

In the studied cohort, 34 % of the patients demonstrated 452 class II imbalances (Fig. 1). This high percentage is most probably due to the fact that the first 250 cases of this cohort of patients were highly selected as they had a strong clinical phenotype, normal karyotype and most had a normal subtelomeric analysis by fluorescence *in situ* hybridization (FISH) or multiplex ligation-dependent probe amplification (MLPA). Patient selection is one of the factors that most influences the diagnostic yield, as stated by Shoukier and collaborators, whose cohort of patients had already performed conventional cytogenetics and subtelomere screening before array-CGH, similar to our first 250 patients [12].

Almost 50 % of the identified class II imbalances were inherited, making the genotype/phenotype correlation difficult to establish as most of the carrier progenitors had apparently normal phenotypes, although they have not been through a thorough clinical evaluation. As previously mentioned, larger inherited imbalances also had a phenotypic manifestation in the carrier progenitor. Smaller inherited imbalances can either not be the cause of the proband’s phenotype or can have incomplete penetrance in the carrier progenitor. There are some exceptions, as for example the 12.5 Mb inherited imbalance that corresponds to an X-chromosome duplication in a boy, inherited from his healthy mother, which illustrates how X-chromosome inactivation in females can mask large imbalances. In the range of 8–9 Mb we identified a chromosome 4 maternal duplication in a girl with attention-deficit hyperactivity disorder whose mother presented learning difficulties at school [17]. In the 6–7 Mb range two imbalances were identified: in 18p11.32p11.31 with 6.9 Mb duplication and in 8p21.3p21.1 with 6.2 Mb duplication both inherited from affected fathers, one with ID and the other with learning difficulties, as he only completed basic school.

Class II imbalances have a spectrum that ranges from pathogenic, potentially pathogenic to uncertain significance but likely pathogenic (Table 1). In this type of imbalance, the study of parents is recommended and helps the prioritization of CNVs – if *de novo*, CNV is most likely pathogenic.

### Class IIIA - Deletion or duplication reported in low frequency in the DGV or that do not totally overlap with the ones reported, involving known coding genes

Imbalances classified in this class corresponded to 20 % of all the identified CNVs (Fig. 1). These CNVs can be population CNVs with low representation in the public databases. This class includes imbalances of uncertain significance tending to be probably benign. In only one patient inheritance was determined to be *de novo*, and in this patient the imbalance might be considered of uncertain significance but likely pathogenic.

Class IIIA imbalances have uncertain significance, ranging from likely pathogenic to likely benign (Table 1). In this type of CNVs, the study of parents is optional and the management of the origin and its impact has to be evaluated in the clinical context.

### Class IIIB - Deletion or duplication reported in low frequency in the DGV or that do not totally overlap with the ones reported, without known coding genes

In this cohort of patients, 22 % of the imbalances were classified as IIIB. Accordingly to the available data, as they do not include genes, these imbalances were not considered relevant for the phenotype. Nevertheless, they were included in a table attached to the report sent to the clinician, allowing these cases to be revisited and reclassified in the light of future knowledge.

Class IIIB imbalances have uncertain significance, but being likely benign (Table 1). The study of parents is not recommended.

## Conclusion

Array-CGH has revolutionized post natal cytogenetics diagnostic, increasing the detection rate and replacing conventional cytogenetics [1]. However, karyotyping cannot be completely abandoned and, in certain cases, both techniques should be performed in order to understand the biological mechanism involved in the origin of an imbalance and contribute, not only to identify the carriers, but also to do a correct evaluation of the recurrence risk in these families. The importance of the proposed CNVs prioritization arises, specially, when a preliminary report is done requesting parental studies. It was important for the clinical management of the family to have an indication of the relationship between the genotype and the phenotype. Classifying CNVs in different classes allowed us to establish the continuum pathogenic–benign and their final impact on the phenotype, after determining their origin. In classes I and II, parental blood samples were always requested to determine the origin of the imbalance either by array-CGH, MLPA, FISH or karyotype. Prenatal diagnosis was recommended in subsequent gestations to progenitors from classes I and II probands. In class IIIA imbalances it was left to clinicians’ criteria, after the genetic counseling, the necessity to perform subsequent laboratory and familial studies. For classes IIIB and IV no parental blood samples were requested by the laboratory, but these results were sent to the clinician in a table attached to the report for future management.

This classification allowed a prioritization of the cases, particularly when a report had to be issued requesting parents study.

## Methods

### Patients

A cohort of 1000 unrelated patient samples was analyzed by oligoarray-CGH in our Cytogenetics and Genomics laboratory over a period of 3 years. The samples were provided by clinical geneticists and pediatricians from 3 national hospitals, as part of a common undertaking to determine the etiology of ID with or without dysmorphisms, learning difficulties, MCA and ASD. The levels of ID of our patients ranged from mild to moderate or severe as determined by the gold standard scales and the ASD patients were evaluated by ADI-R and ADOS. The study was approved by the Hospitals Ethics Committee.

Various techniques were used to confirm and characterize the imbalances identified by oligoarray-CGH, such as, high resolution conventional cytogenetics, FISH or MLPA, depending on the type and dimension of the imbalance and availability of FISH and MLPA probes in the laboratory. In order to determine the origin of the imbalances, parental blood samples were requested and, additionally to the previous mentioned techniques, array-CGH was also used in particular cases.

In 325 patients conventional cytogenetics had been previously performed. Fourteen patients with an abnormal karyotype were analyzed by array-CGH. Of those, five patients presented a deletion, two a duplication, two had apparently balanced translocations, one had a 47, XXY karyotype, three patients presented inversions and one had a derivative chromosome. These cases were analyzed by oligoarray-CGH to refine the breakpoints and the genomic content of the rearrangements, or to determine if there were genomic imbalances involved in apparently balanced translocations, as they can remain undetected [18], and inversions carriers, or even to clarify if there was an additional imbalance, like in the case of a Klinefelter patient, which could explain a severe phenotype.

### DNA extraction

Genomic DNA was extracted from peripheral blood lymphocytes using Jetquick blood and cell culture DNA Midi Spin kit according to the manufacturers’ instructions (Genomed, Löhne, Germany). DNA concentration and purity were measured using a NanoDrop1000 Spectrophotometer (Thermo Scientific, Waltham, USA).

### Array-CGH

High-resolution whole genome analysis was performed using Agilent SurePrint G3 Human Genome microarrays. Although 180 K oligonucleotide microarray format (Agilent Technologies, Santa Clara, CA, USA) was the most used, the 60 K was requested for fifty-four cases. These microarrays contain approximately 180 000 and 60 000 sixty-mer probes with a 17 kb and 54 kb average probe spacing, respectively. Labeling was performed using Agilent Genomic DNA enzymatic labeling kit (Agilent Technologies, Santa Clara, CA, USA) and clean-up of labeled genomic DNA was performed using Amicon ultra 0.5 ml centrifugal filters according to manufacturers’ instructions (Millipore, Billerica, MA, USA). Slides were scanned on an Agilent scanner and processed with Feature Extraction software (v10.7). Each patient DNA was labeled both in Cy3 and Cy5 and hybridized against the DNA of two phenotypically distinct patients according to the loop model [19]. Results were analyzed using Agilent Genomic Workbench (v6.0 and v6.5) software with the following settings: ADM2 as aberration algorithm, threshold of 6.0, moving average 2 Mb. The results are according to Human Genome build 19 and include imbalances with at least three consecutive probes with abnormal log_2_ ratios. All the imbalances were interpreted consulting the UCSC genome browser (http://genome.ucsc.edu), Decipher (Database of Chromosomal Imbalance and Phenotype in Humans Using Ensembl Resources - http://decipher.sanger.ac.uk/), ClinGen (Clinical Genome Resource - https://www.clinicalgenome.org/), OMIM (Online Mendelian Inheritance in Man - http://www.ncbi.nlm.nih.gov/omim) and DGV (http://projects.tcag.ca) databases.

### Statistical analysis

The statistical analysis was mainly conducted aiming to describe the proportions found in each group resorting to suitable figures and tables. Association hypotheses regarding nominal variables were tested using the chi-square test taking into account the Cochran rules for validity of the test. The level of significance adopted was 0.05 %.
